# A Crab Is Not a Fish: Unique Aspects of the Crustacean Endocrine System and Considerations for Endocrine Toxicology

**DOI:** 10.3389/fendo.2021.587608

**Published:** 2021-03-02

**Authors:** Thomas Knigge, Gerald A. LeBlanc, Alex T. Ford

**Affiliations:** ^1^Normandy University, FR CNRS 3730 SCALE, UMR-I 02 INERIS-URCA-ULH Environmental Stress and Aquatic Biomonitoring (SEBIO), Université Le Havre Normandie, Le Havre, France; ^2^Department of Biological Sciences, North Carolina State University, Raleigh, NC, United States; ^3^School of Biological Sciences, Institute of Marine Sciences, University of Portsmouth, Portsmouth, United Kingdom

**Keywords:** endocrine disruption, neuroendocrine disruption, ecdysteroid signaling, color change, sexual differentiation

## Abstract

Crustaceans—and arthropods in general—exhibit many unique aspects to their physiology. These include the requirement to moult (ecdysis) in order to grow and reproduce, the ability to change color, and multiple strategies for sexual differentiation. Accordingly, the endocrine regulation of these processes involves hormones, receptors, and enzymes that differ from those utilized by vertebrates and other non-arthropod invertebrates. As a result, environmental chemicals known to disrupt endocrine processes in vertebrates are often not endocrine disruptors in crustaceans; while, chemicals that disrupt endocrine processes in crustaceans are often not endocrine disruptors in vertebrates. In this review, we present an overview of the evolution of the endocrine system of crustaceans, highlight endocrine endpoints known to be a target of disruption by chemicals, and identify other components of endocrine signaling that may prove to be targets of disruption. This review highlights that crustaceans need to be evaluated for endocrine disruption with consideration of their unique endocrine system and not with consideration of the endocrine system of vertebrates.

## Introduction

Over the last two decades, both the scientific community and the lay public have become increasingly aware of the risk associated with endocrine disruption. Environmental authorities in Europe, the United States and many other countries are preoccupied with the identification of Endocrine Disrupting Chemicals (EDCs) and their effects on human health and wildlife. In 2012, a report on the state of endocrine disruption was composed under the auspices of the United Nations Environment Program (UNEP) and the World Health Organization (WHO). This report stated that in spite of numerous reports of reproductive disorders, skewed sex ratios and intersex phenomena, the understanding of endocrine disruption in invertebrates—including crustaceans—was limited (1). Since then some progress has been made, but knowledge about the effects of EDCs on the endocrine system of crustaceans remains scarce and concerns mainly pesticides designed to disrupt moulting in insects (2, 3). Among the vast array of chemical compounds listed on the Toxic Substances Control Act (TSCA) inventory (US EPA)—and many others not regulated under the TSCA—there will certainly be many other chemicals that have the potential to interfere with the crustacean endocrine system.

With about 68,000 extant species of crustaceans described—and still more to be discovered—these predominantly aquatic invertebrates form a large and very diverse arthropod taxon (4–6). As predators, scavengers, or filter feeders, they take important positions within the aquatic ecosystems at various levels of the food web. They also provide high-value fishery products and contribute with over 14 million tons—of which half are wild stock captures—to about 8% of the worldwide seafood resources (7).

This ecological and economical wealth is, however, endangered by habitat loss, climate change, pollution, overexploitation, invasive species and other anthropogenic stressors (8). In the early nineties, drastic declines of amphipods were reported in some of the Great Lakes (9, 10). Similarly, populations of the amphipod *Gammarus lacustris* significantly declined in the Selenga River delta, the main tributary to Lake Baikal (11). Although pollution, such as polychlorinated biphenyls (PCBs) or pulp mill effluents, respectively, were given consideration, the actual cause-effect relationships were complex and difficult to establish. Massive decreases in blue crab populations of Chesapeake Bay occurred in the nineties, where spawning stock abundance declined by about 80% (12). Similarly, catches of the edible crab, *Cancer pagurus*, in the English Channel were halved from 2012 to 2018 with no clear cause for this decline (13). Even if overfishing, increasing temperatures, and ocean acidification are contributing causes, reproduction impairment due to endocrine disruption could be adding to the declines, or hampering the recovery of stocks. A contribution of EDCs in these declines of marine and freshwater crustaceans is difficult to discern. Firstly, it is generally challenging to determine the quantitative contribution of individual challenges to population sustainability. Secondly, it is difficult to identify endocrine disruption without knowing how to measure it. In any case, no prominent example of endocrine disruption, comparable to imposex in prosobranch gastropods, is known for crustaceans. Examples of intersex that appear to be related to pollution have been reported for different crustacean species (14–19). But no cause-effect relationships have been established.

The assessment of endocrine disruption in crustaceans can be approached either from the perspective of the molecules suspected to cause endocrine disruption, *i.e.*, effects of established EDCs, or from the perspective of the endocrine targets of EDCs, *i.e.*, crustacean endocrinology. Environmental chemicals that have been classified as EDCs, are, for the most part, compounds that interfere with the vertebrate hormone system. Strenuous effort has been made to demonstrate possible endocrine disrupting effects of such EDCs in crustaceans (20–30). Although effects on growth and reproduction are often reported, most studies failed to demonstrate that endocrine mechanisms were involved. The generally high concentrations that are necessary to produce negative consequences for growth or reproduction suggest that the observed effects were merely a result of overt toxicity (*e.g.*, (31, 32). More specific assays are being developed that use elements of the crustacean endocrine system, such as reporter assays for the ecdysteroid and methyl farnesoate receptors, measurement of 20-hydroxyecdysone (20E) titers, or assays for chitobiase activity (33–38). Nevertheless, establishing reliable testing protocols for endocrine disruption in crustaceans remains challenging and widely ignores the importance of neurohormonal regulation for the control of many physiological functions in crustaceans.

This review argues in favor of a more arthropod specific approach to endocrine disruption in crustaceans.

## Evolution of the Invertebrate Endocrine System

Endocrine systems are key features of evolution reflecting metazoan diversification (39). Metazoan endocrine systems have evolved from a common bilaterian ancestor before the divergence of protostomes and deuterostomes more than 600 million years ago (40, 41). Specialized neurosecretory cells were already present in the pre-bilaterian cnidarians and neurohormonal signaling persists as a major endocrine component in both, the protostome and deuterostome lineages (42–44). The arthropods diverged within the protostome lineage some 500 million years ago (45). Unique aspects to the physiology of the arthropods, including crustaceans, required the development of unique endocrine pathways to regulate these physiological processes. For example, arthropods lost the capacity to synthesize cholesterol (41, 46). This loss of cholesterol synthesis may have limited opportunities for the evolution of steroid hormones, which utilize cholesterol as a precursor. However, this loss may have also promoted the evolution of methyl farnesoate (MF) in crustaceans and juvenile hormone in insects, which do not utilize cholesterol, into functional hormones (41). The divergence of the arthropod endocrine system has been deepened further by the evolution of an exoskeleton in arthropods, which requires moulting for growth and reproduction (47). Endocrine signaling processes were required to regulate moulting and coordinate this process with those operative in growth and reproduction.

While the endocrine systems of the two major arthropod taxons, crustaceans and insects, share many commonalities, divergences also occurred. For example, both groups possess the capacity to produce MF. However, insects possess a cytochrome P450 monooxygenase, which epoxidates MF to form juvenile hormone III (48). This latter hormone regulates many of the processes in insects that are under the control of methyl farnesoate in crustaceans. Crustaceans produce a family of hormones known as crustacean hyperglycemic hormones (CHHs) (49, 50). These hormones regulate a myriad of processes including aspects of moulting, reproduction, and energy generation. Only a single member of this family, the ion transport peptide, is known to exist in insects. This insect hormone controls ion transport (51, 52).

The presence or absence of nuclear receptors, *i.e.*, ligand-regulated transcription factors, reflects the separation of steroid hormone signaling within the endocrine systems of crustaceans and vertebrates. The evolution of these receptors was shaped by whole genome duplications and losses combined with the evolution of neofunctionalization or subfunctionalization among duplicate receptors (39, 53–55). Importantly, arthropods lack receptors of the steroid receptor subfamily 3, which include the estrogen and androgen receptors. This may represent the loss of the progenitor of this subfamily (56, 57). However, subfamily 3 receptors are also absent in the ascidian *Cionia intestinalis*, a deuterostome invertebrate distantly related to vertebrates (55, 58). This latter observation suggests that subfamily 3 nuclear receptors evolved later in chordate evolution, long after protostomes and deuterostomes diverged (59, 60). This hypothesis is supported by the appearance of sex steroid receptors, including the estrogen receptor, in the cephalochordate *Amphioxus* (61, 62). The ecdysteroid receptor (EcR) of arthropods does not belong to the subfamily 3 nuclear receptors, but is an ortholog of the vertebrate liver X and farnesoid X receptors, which are members of the subfamily 1 nuclear receptors (63).

The loss and gain of functions and elements of the endocrine system, such as nuclear receptors or neuropeptide hormones in arthropods is likely to be related to the invention of a new body plan with an exoskeleton (64). The exoskeleton constrains growth and the impermeable cuticle limits gas and ion exchange with the environment. Moulting solves the first problem, while the second problem requires organs for gas (and ion) exchange, such as the gills in crustaceans or the tracheae in insects, both of which have similar organogenetic origins. Sanchez-Higueras et al. (65), demonstrated that trachea develop as serial homologues to the ectodermal prothoracic gland and the *corpora allata*, which have their counterparts in the Y-organ and the mandibular organ of crustaceans (see also (43)). These endocrine glands produce ecdysone and juvenile hormone/MF, respectively, *i.e.*, insect/crustacean hormones related to moulting and metamorphosis. Hence, these findings link the evolution of arthropod hormones and endocrine organs with the evolutionary innovation of an exoskeleton. As a matter of consequence, the homology of endocrine organs and gills provides evidence for the integration of environmental cues into physiological responses, such as respiration, osmoregulation and growth by an endocrine system specific to arthropods.

Crustaceans and vertebrates share the principal concept of connecting neural, neurosecretory, and endocrine components (58). Yet, the endocrine systems of arthropods and vertebrates have evolved independently and differently since more than 540 million years ago when crustaceans and deuterostomes appeared in the early Cambrian (66, 67). As a matter of consequence, they have little in common. The fundamental difference between arthropod and vertebrate endocrine systems has implications for determining endocrine disruption. Exogenous substances that alter functions of the endocrine system are, for the most part, likely to be different for crustaceans and vertebrates. Substances designed to interfere with the insect endocrine system (*i.e.*, insect growth regulating insecticides) by their design have significant potential to interfere with the crustacean endocrine system, but are much less likely to interfere with the endocrine system of vertebrates. Conversely, steroid hormone functional analogs are highly likely to disrupt endocrine regulated processes in vertebrates, but are much less likely to disrupt endocrine signaling in crustaceans. The most conserved elements associated with the endocrine regulation of physiological functions shared by both, arthropods and vertebrates are the upstream control of neurosecretory processes by biogenic amines. Besides their function as neurotransmitters and neuromodulators, biogenic amines, such as serotonin, can also serve as neurohormones circulating in the blood stream and affecting peripheral organs like ovaries.

## Endocrine Regulation Of Moulting

**Figure 1:**
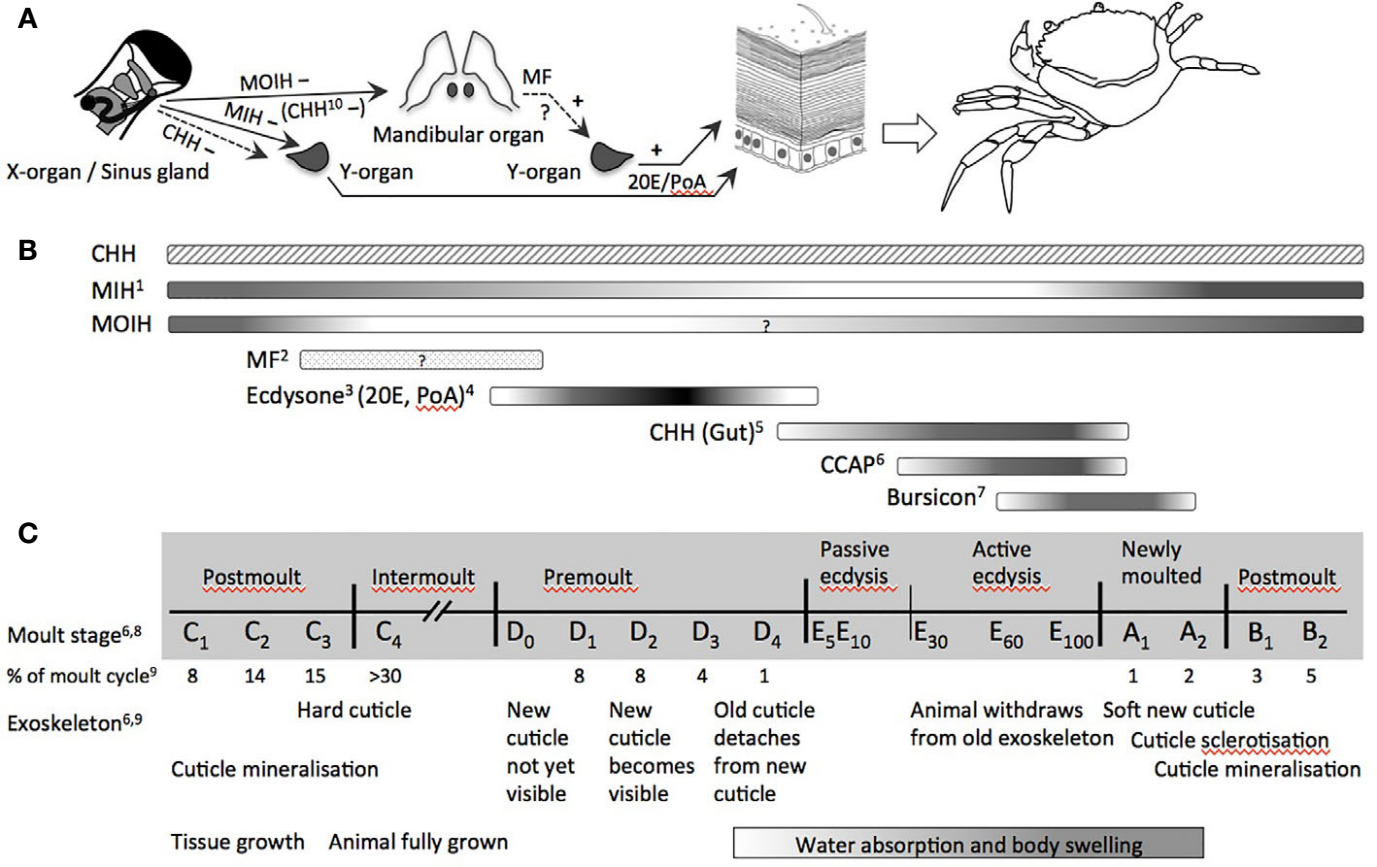
Endocrine control of moulting in decapod crustaceans (Malacostraca). **(A)** Organs directly involved in the control of moulting (release of CCAP and bursicon by the central nervous system and/or the pericardial organ as well as release of CHH by paraneurons of the fore- and hindgut not shown): MIH and CHH from the X-organ/sinus gland-complex negatively inhibit the synthesis of ecdysone by the moulting gland (Y-organ); MOIH (and in some species CHH) negatively inhibit the synthesis of MF, the stimulatory activity for ecdysteroid synthesis has been reported in several studies; ecdysteroids of the major forms 20E and PoA stimulate the epidermis *via* its corresponding EcR to decalcify and to lyse the membrane layer and the old cuticle by proteases, chitinase and chitobiase, and to synthesize material for the new cuticle. **(B)** Dynamic hemolymph titres of (neuro-)hormones involved in the control and/or physiological processes during moulting and ecdysis over an entire moult cycle. **(C)** Moult stages and main modifications of exoskeleton and growth (non-proportional presentation with respect to duration of each period). Abbreviations: CCAP, crustacean cardioactive peptide; CHH, crustacean hyperglycemic hormone; EcR, ecdysteroid receptor; MF, methyl farnesoate; MIH, moult-inhibiting hormone; MOIH, mandibular organ-inhibiting hormone; 20E, 20-hydroxyecdysone; PoA, ponasterone **(A)** ^1^ (70); ^2^ (71); ^3^ (72); ^4^ (73); ^5^ (74); ^6^ (75); ^7^ (76); ^8^ (77); ^9^ (78); ^10^ (79).

The monophyly of arthropods is based on a sclerotized exoskeleton (68). To allow for growth, the cuticle has to be shed and renewed periodically. Moulting, *i.e.*, ecdysis, is a distinctive characteristic of the crustacean life cycle (69), which involves a complex interplay of numerous neuropeptide and steroid hormones (**Figure 1**), thereby covering key features of the endocrine system of crustaceans. As such it is an endpoint par excellence for endocrine disruption in crustaceans. In many cases, moulting in crustaceans is also related to reproductive periods by alternating cycles of moulting and reproduction, as in *Daphnia* (80, 81), so that perturbations of ecdysis, may also affect reproductive phases.

The crustacean moult cycle is divided into five major stages comprising numerous substages [**Figure 1**; (69, 82)]. During the intermoult stage (C_4_), the neuropeptide moult-inhibiting hormone (MIH) represses ecdysteroid synthesis. The proecdysial period begins with the apolysis of the old exoskeleton, during which the membrane layer and the endocuticle are degraded and their building materials are partially resorbed (D_1_–D_3_). Simultaneously, the new exoskeleton is synthesized. A surge of ecdysone during premoult triggers downstream events that lead to the extrication of the animal from its carapace (E). Despite the long history of endocrine research in crustaceans, the hormonal regulation of these events still lacks precise knowledge of some factors, such as homologs of ecdysis-triggering hormone (ETH) and eclosion hormone (EH). The function of these neuropeptides is well described for insects, but awaits further elucidation in crustaceans. More recent work also suggests a yet unknown role for corazonin in the control of moulting (83). Eventually, CHH and crustacean cardioactive peptide (CCAP) are tightly linked, respectively, with ecdysial water uptake and the onset of the behavioral motor program by which the animal extricates from its carapace. Tanning and sclerotization of the new cuticle are under the control of bursicon, another neuropeptide hormone. At large, the complex control of moulting described in the following, which involves many other hormones beyond MIH and ecdysone, provides various targets for endocrine disruption in crustaceans.

### Neuro-Endocrine Regulation

Almost 70 years after Passano (84) proposed the existence of MIH secreted by the major neurohemal organ in decapod crustaceans, the X-organ/sinus gland (XO/SG) complex of the eyestalk (reviewed in (85, 86)) and the characterization of the respective neuropeptide by Webster and Keller in 1986 (87), the main features of neuroendocrine moult control are generally accepted for decapods. According to this regulatory model, the activity of the moulting gland (*i.e.*, Y-organ) is inhibited by MIH during intermoult, thus subjecting ecdysteroid signaling to the negative control by neuropeptides from the XO/SG (**Figure 1**) (69, 72, 88–90). Evidence is provided by experiments removing eyestalks or XO/SGs, which results in shortened moult intervals and higher moult frequency (72). This inhibition of ecdysis in eyestalk/XO/SG-ablated animals can be restored with extracts from the SG. Dynamic variations of MIH during the moult cycle as well as decreases of MIH stored in the SGs and circulating MIH in the hemolymph during pro- and postecdysis further corroborated the role of MIH in neuro-endocrine regulation of ecdysis (70, 88, 90, 91). Furthermore, Techa and Chung (70) suggest a feedback control by which elevated ecdysteroid concentrations in the hemolymph stimulate *mih*-expression, but inhibit MIH-secretion. As a matter of consequence, high amounts of MIH are stored in the SG during ecdysis and released during post-moult, resulting in low ecdysteroid titers. Hence, any perturbation of synthesis, storage and release of MIH from the XO/SG could result in modifications of Y-organ inhibition, ecdysone synthesis and the resulting circulation of ecdysteroid levels (*e.g.*, (92)).

MIH is not the only factor controlling ecdysteroid secretion from the Y-organ. During proecdysis, the Y-organ becomes progressively less sensitive toward MIH-mediated inhibition of ecdysteroid production (88, 89, 93, 94), most likely due to modifications in intracellular signaling cascades following binding of MIH to its G-protein coupled receptor. Little attention has been given to the role of CHH in the inhibition of ecdysone synthesis. Albeit, CHH is 10-fold less effective in inhibiting the Y-organ, its levels are roughly 10-fold higher than those of MIH. Chung and Webster (88), therefore, argued that CHH could have an equivalent role to MIH in the negative control of ecdysteroid synthesis. The situation is further complicated by the implication of other neurohormones in moulting (**Figure 1**). Notably, CCAP displays considerable dynamics during ecdysis (75) and increases massively during late ecdysis when the animals actively exuviate. Phlippen et al. (75) explain this surge of CCAP by its mytropic action, thus potentiating muscle contraction while the animals withdraw from their old exoskeleton, and by its effect on cardiovascular functions regulating hemolymph flow and pressure. Interestingly, CHH released from paraneurons of the fore- and hindgut rises about 100-fold during ecdysis, followed by the surge of CCAP, both reaching their maximum at late ecdysis and decreasing rapidly in newly moulted animals (74) (**Figure 1**). Injections of CHH at physiological concentrations were able to initiate ecdysis through swelling as a result of isosmotic water uptake by the hindgut, suggesting a role of CHH in ion and water uptake during moulting, similar to the function of the homologous ion transport peptides in the hindgut of insects (74). Eventually, bursicon, another neuropeptide hormone produced by the central nervous system overlapping with CCAP is massively released from the pericardial organ into the hemolymph when ecdysis is completed and remains significantly elevated during early postmoult (76) (**Figure 1**). Its consistent presence throughout the moult cycle at about one quarter of its concentration during ecdysis gives rise to further cuticle-associated functions of bursicon during moulting (95).

Interestingly, the nature of hindgut CHH, CCAP, and bursicon and their role in ecdysis bear resemblance to neuropeptides involved in the ecdysis of insects. Because any spontaneous release of, notably, CCAP could be excluded, the existence of yet non-identified ecdysis-inducing factors similar to ETH and EH of insects would complete the overall picture of ecdysis in crustaceans (75). Indeed, analyses of the *Daphnia pulex* genome, suggest that such factors may exist in crustaceans (96, 97). More recently, carcikinin, an ortholog of ETH was identified in *Carcinus maenas* (98) and ETH genes were found in several crustacean transcriptomes [see (99) for references]. Injection of ETH into the crayfish *Cherax quadricarinatus* prolonged the moult period, suggesting a role in the control of the moult cycle in decapod crustaceans (99). Similarly, EH was identified in the shrimp *Exopalaemon carinicauda*, and displayed highest expression levels during premoult, while gene-silencing lead to a delay in moulting and reduced rates of ecdysis (100). Furthermore, the virtually exclusive expression of a G-protein coupled receptor for corazonin in the Y-organ indicates a role for this neuropeptide in the control of ecdysis (83). Despite these promising results, the roles of ETH, EH, and corazonin in crustacean ecdysis await further characterization.

### Ecdysteroids

Ecdysteroids are the predominant hormones responsible for moulting and other processes in crustaceans. Ecdysteroid metabolism is relatively well understood owing to advances in insect biochemistry and conserved pathways between insects and crustaceans. While crustacean ecdysteroids and their nuclear receptors are similar to those of insects, they differ in the number of hormones and in the number and structure of the receptor isoforms (101).

The concentrations of ecdysteroid hormones circulating within crustaceans vary during the moult cycle, and depending on the species, either gradually or quite rapidly spike to start a period of ecdysis or moulting (**Figure 1**) (102). Ecdysteroids and the enzymes responsible for breakdown of the chitin skeleton have been measured through various molecular and cellular assays targeting gene expression and/or enzyme synthesis, respectively.

#### Enzyme Synthesis and Inactivation

Ecdysteroid biosynthesis is divided into two stages (103). The first stage involves the conversion of cholesterol, derived from diet, to 5β-diketol and the second stage converts 5β-diketol to secreted products. Depending on the species, four major secreted products are ecdysone, 3-dehydroecdysone (3DE), 25-deoxyecdysone (25dE), and 3-dehydro-25-deoxyecdysone (3D25dE) (103). A large number of ecdysteroidogenic enzymes and associated genes have been identified in insects and other arthropods (104) with a large degree of conservation in the crustaceans (105, 106). For example, orthologs of *nmg*/*sro*, *spo*, *phm*, *dib*, *sad*, and *shd* have been identified in the *D. pulex* genome (46, 107, 108).

Both the increase and subsequent rapid decline in ecdysteroid titers are critical to moulting. The cytochrome P450 hydroxylase cyp18a1 is primarily responsible for the inactivation of the hormone, rendering it susceptible to further modification and elimination (103, 109).

The inhibition of cytochrome P450s by chemical compounds is a widespread mechanism (110), which could interfere with the multiple hydroxylation reactions catalyzed by the cytochrome P450s encoded by the “Halloween genes” (104). For instance, several classes of fungicides function by inhibiting cytochrome P450-mediated demethylation of sterols that are critical components of fungal cell membranes. Some of these compounds are also capable of inhibiting cytochrome P450s of non-target organisms that are involved in steroid biosynthesis. Exposure of *Daphnia magna* to the demethylase inhibiting fungicides fenarimol, pyrifenox, prochloraz, triadimefon, and propiconazole delayed moulting and/or caused developmental abnormalities in neonates (111, 112). Mechanistic studies revealed that fenarimol exposure reduced ecdysteroid levels in daphnids and that co-exposure with 20E protected against the delay in moulting and developmental abnormalities caused by this fungicide (113). These results are consistent with the hypothesis that fenarimol elicited toxicity by inhibiting cytochrome P450s involved in ecdysteroid synthesis.

#### Receptor-Mediated Activation

Ecdysteroid hormones, such as the major forms 20E and ponasterone A (PoA), bind to the ligand-binding domain of the EcR and activate the expression of primary early ecdysteroid responsive genes, such as E75 and E74 and early late genes such as HR3 and HR4 (114, 115) (see also reviews by (116, 117)). These transcriptional regulators drive the “late genes” responsible for metamorphosis, moulting, and/or ovarian development (114, 118–120). For ligand binding, the EcR forms a heterodimer with the retinoid-X-receptor (RXR), the ortholog of the insect ultraspiracle (121). The RXR contributes to DNA-binding and helps to stabilize the EcR-ligand binding pocket and allows for flexibility in ligand binding (122, 123). Unliganded EcR-RXR is a repressor of transcription (123).

An account of the transcriptional activities of some of these late genes during premoult and ecdysis is given by Li et al. (124). Notably, genes involved glucosamine synthesis, corresponding to the formation of material for a new cuticle, were upregulated during the premoult, whereas genes encoding chitin synthase and several chitinases were upregulated during ecdysis and postmoult stages. Consequently, enzymes such chitobiase (125), N-acetyl-β-glucosaminidase (126) or chitin synthase (127) can be used as biomarkers for EcR-induced late genes provided that the xenobiotic concentrations are low and act *via* EcR-signaling rather than inhibiting the enzyme itself or exerting non-specific effects on gene-expression. Furthermore, enzyme-expression and activity have to be put in the perspective of the precise moulting stage (127, 128).

### Disruption of Ecdysteroid Signaling

Several targets exist at which environmental chemicals might interfere with ecdysteroid signaling. These include disruptions in ecdysteroid synthesis, ecdysteroid inactivation, and interactions with the EcR. The neuroendocrine regulation of ecdysteroid signaling also may provide targets of disruption, though few definitive examples of such disruption exist. Overall, the XO/SG-Y-organ-EcR axis of malacostracan crustaceans offers an endocrine signaling cascade similar to the mammalian hypothalamus-pituitary-gonadal-ER axis.

#### Modulation of Ecdysteroid Levels

Studies have reported on the ability of environmental chemicals to alter the expression of ecdysteroid biosynthetic or biotransformation enzymes. For example, exposure of the copepod *Tigriopus japonicas* to 20 mg·L^–1^ atrazine resulted in a reduction of mRNA transcripts for several enzymes involved in ecdysteroid biosynthesis and biotransformation (129). Unfortunately, impacts on ecdysteroid levels were not established. A generalized reduction in relevant gene expression levels suggest that atrazine may have disrupted common neuro-endocrine control of these genes, or that the high concentration of atrazine used resulted in overt toxicity presented as an overall decrease in transcription.

Exposure of the Chinese mitten crab, *Eriocheir sinensis*, to the pharmaceutical carbamazepine reduced hemolymph ecdysteroid levels and epidermal chitobiase activity. Carbamazepine exposure also increased *chh* and *mih*-expression; while, decreasing EcR and RXR mRNA levels. Taken together, these effects suggest that carbamazepine may have perturbed the neuroendocrine control of ecdysteroid synthesis resulting in a decrease in ecdysteroid levels and a down-regulation of ecdysteroid-regulated genes. These perturbations in the ecdysteroid-signaling pathway also resulted in delayed moulting (130).

#### Ecdysteroid Receptor Agonists/Antagonists

Plants produce compounds with ecdysteroidal activity, presumably to serve as an endocrine-disrupting defense against invading insects (*e.g.*, (131)). Similarly, EcR-agonist activity has been exploited as a mode of action of some next-generation non-steroidal insecticides (132). The insecticidal EcR-agonist tebufenozide only weakly activated the *D. magna* EcR in a reporter two-hybrid assay (133). Similarly, tebufenozide, along with the related diacylhydrazines halofenozide and methoxyfenozide were weak agonists in a reporter assay containing the shrimp *Neocaridina davidi*-EcR (34). De Wilde et al. (33), however, could not confirm accommodation of tebufenozide into the ligand binding pocket of the shrimp *Neomysis integer* and found no effect of 100 µg tebufenozide·L^–1^ on nymphal development and moulting. Taken together, these results are consistent with the manufacturer’s report that the diacylhydrazine insecticides exhibit low specificity for EcRs of non-target arthropods (132). They may, however, displace 20E and/or PoA from the binding site by competitive binding (34).

A recent *in silico* study by (134) identified 274 potential non-steroidal EcR-ligands. Furthermore, the screening of 8795 compounds listed in the US EPA’s ToxCast chemical library revealed 34 potential agonists including the diacylhydrazines insecticides and numerous pharmaceuticals, such as non-steroidal anti-inflammatory drugs containing pyrazolone derivatives, or members of the amphenicol antibiotic family. Using the *Drosophila melanogaster* B_II_ cell assay, Dinan et al. (135) detected no EcR-agonist activity among 80 environmental chemicals. Bisphenol A, diethylphthalate, some polycyclic aromatic hydrocarbons, naphthalenes, pesticides, and pharmaceuticals were weak antagonists in this assay. Notably, estradiol, progesterone, and testosterone as well as synthetic steroids neither displayed agonist, nor antagonist activity, except for two compounds, 4-androstene-3,17-dione and 17α-ethinylestradiol, which were weak antagonists.

Despite the lack of anti-ecdysteroidal activity associated with testosterone reported by Dinan et al. (135), weak EcR-antagonist activity of testosterone was reported in daphnids (136). Exposure of *D. magna* to micromolar concentrations of testosterone caused a concentration-dependent delay in moulting and an increase in developmental abnormalities among neonates. Co-exposure with 20E protected against this toxicity of testosterone. Testosterone did not lower endogenous 20E-levels, but rather appeared to antagonize the EcR, based upon competition assays between testosterone and 20E in ecdysone-responsive *Drosophila* Kc cells. While these results suggest that testosterone is anti-ecdysteroidogenic in daphnids, the results have little environmental relevance to environmental androgens due to the high concentrations required to elicit a response.

#### Gene Product Changes

Several studies have reported on the impacts of exposure to pollutants, pharmaceuticals, and vertebrate hormones on expression profiles of mRNAs or proteins along the ecdysteroid signaling pathway. Expression of *ecr* was elevated from exposure of prawns, *Macrobrachium potiuna*, to glyphosate-based herbicides, ethinylestradiol (estrogen), 4-hydroxytamoxifen (anti-estrogen), 17α-methyltestosterone (androgen), and cyproterone acetate (anti-androgen) (30, 137). Exposure of the intertidal mud crab *Macrophthalmus japonicas* to bisphenol A and di-(2-ethylhexyl) phthalate significantly elevated *ecr* expression levels (138). Studies, such as these are indicative of exposure to potential endocrine disrupting chemicals. However, whether the exposure actually results in apical disruption remains equivocal in the absence of demonstrated consequences of the molecular alterations.

Conversely, several studies have reported on effects of chemical exposure on apical endpoints, such as moulting. Zou (139) identified 33 compounds that have been shown to delay, impede, or advance moulting in crustaceans. While such studies inform on the toxicity of the chemicals to crustaceans, they do not provide insight on whether the effects elicited are actually due to endocrine disruption.

#### Neurohormone Activity Modulation

The negative control of ecdysteroid synthesis by MIH and by CHH is under the control of biogenic amines, notably, serotonin (5-hydroxytryptamine, 5-HT). Evidence suggests that the hyperglycemic action of 5-HT is due to the direct stimulation of CHH neurons (50, 140, 141). Indeed, 5-HT-immunopositive efferent axons to the *medulla terminalis* and the XO-neuropile have been demonstrated (142). The excitatory role of 5-HT on these XO-neurons was shown by Sáenz et al. (143). Therefore, serotoninergic stimulation of the release of neurohormones from the XO/SG-complex is likely to be a general phenomenon that applies to CHH as well as to MIH or mandibular organ inhibiting hormone (MOIH). The effects of fluoxetine on ecdysteroid levels in *C. maenas* demonstrated that fluoxetine, a selective serotonin reuptake inhibitor, significantly decreased 20E-levels at 0.5 and 0.75 µM after 8 and 4 h, respectively (92). Because, this effect was even more rapid and more pronounced with 0.5 µM 5-HT, but no effect of 5-HT or fluoxetine on 20E-levels could be observed in eyestalk-ablated animals, it was concluded that the mechanism leading to reduced 20E would be the inhibition of ecdysteroid synthesis by 5-HT-stimulated release of MIH. Interestingly, low ecdysteroid levels appeared to relate to an increased *mih*-expression in this study.

To date, only few studies examined the effects of pollutants on neuroendocrine processes that control moulting in crustaceans and only recently has MIH been proposed as a biomarker of endocrine disruption in crustaceans (30). This may be explained by the difficulties to quantify MIH-levels in the hemolymph, due to its 10 times lesser concentrations as compared to CHH and because of the pulsatory release of these neuropeptides from the SG (88, 144). Thus, *mih*-expression has been used to evaluate the effects of EDCs on the neuroendocrine regulation of moulting (30, 92, 137). Gismondi (30) and de Melo et al. (137) found an over-expression of *mih* in response to estrogen agonists and antagonists as well as antiandrogenic and androgenic compounds and a glyphosate-based herbicide. Because 20E equally increased *mih*-expression in the study of Gismondi (30), probably by the feedback control described by Techa and Chung (70), it was concluded that EDCs interfering with vertebrate steroid hormone signaling could affect *mih*-expression *via* an ecdysteroid related pathway, but without any further mechanistic explanation. The fact that very different compounds, which either activate or block the estrogen receptor or interact with other steroid receptors and even glyphosate all stimulated *mih*- as well as *ecr*-expression rather points to other, non-specific effects. Nevertheless, increased expression of *mih* may lead to an increased synthesis and release of this neuropeptide with the potential to modulate ecdysis.

### Vertebrate-Type Sex Steroids

Whether or not crustaceans utilize estrogen, androgen, and progestogen signaling pathways has been debated for decades (145). Evidence in support of these signaling pathways is based largely upon observational studies; while, evidence against the existence of these signaling pathways is supported by genomic investigations and evolutionary biology. Interaction with the EcR is often cited as a mechanism by which vertebrate-type sex steroids function in crustaceans. However, as discussed above such interactions typically occur at high, non-physiologic levels.

#### Evidence in Support

##### Presence of the Hormones

The detection of vertebrate-type sex steroids in crustaceans was often cited in earlier literature as evidence that these hormones are of physiological significance in these organisms (146, 147). These include 17β-estradiol and testosterone in amphipods (148) and crayfish (149), pregnenolone in brine shrimp (150), and progesterone in shrimp (151). Several studies report that vertebrate-type steroid levels vary with the ovarian development cycle suggesting some functionality related to this process (discussed in (152)). However, the mere presence of a hormone in an organism does not indicate that the chemical possesses a signaling role in that organism. The hormone may be present as a consequence of dietary uptake or as a non-functional intermediate or metabolite of a biosynthetic pathway (153–156).

##### Responses to Exogenous Steroids

Many studies have demonstrated physiological responses of crustaceans to exogenously administered steroid hormones. For example, administration of 17β-estradiol advanced ovarian development (29) and stimulated vitellogenesis in female decapods (28). This compound also suppressed vitellogenesis-inhibiting hormone gene-expression, which was presumably responsible for the effects on ovarian development (28). While 17β-estradiol administration to crayfish stimulated vitellogenin-mRNA accumulation in the hepatopancreas, progesterone administration increased vitellogenin protein levels in the hemolymph of crayfish (157).

The provision of exogenous 17β-estradiol and progesterone support suggestions of a role for these hormones in crustacean reproduction. Some studies, however, indicate that estrogens downregulate monoamine oxidase activity (158–160) and may, therefore, increase 5-HT-levels that directly influence ovarian development. Furthermore, administration of testosterone has largely resulted in detrimental effects. Administration of testosterone to water fleas suppressed embryo development (136) and decreased lipid storage (161). These effects were attributed to the ability of testosterone to elicit anti-ecdysteroidal activity.

##### Responses to Endocrine Disruptors

Several investigators have reported on the negative effects of estrogenic and anti-androgenic compounds on crustaceans. Estrogenic compounds, such as diethylstilbestrol, endosulfan, Aroclor 1242, and diethylphthalate delayed moulting in *D. magna* (162, 163). Studies that have shown neuroendocrine disruption by some of these compounds [**Table 1**; (172, 173, 176–178)] may provide an explanation for these observations and would situate their effects upstream of ecdysteroid signaling.

**Table 1:** Disruption of neuroendocrine pathways in crustaceans.

Species	Chemical	Concentration*	Effect	Endpoint	Reference
*Procamburus clarkii*	CdCl_2_	5 mg·L^–1^	Stimulation of CHH release; reduction of CHH responsiveness	Glycaemia	(164)
*Uca pugilator*	CdCl_2_	10 mg·L^–1^	Inhibition of PDH synthesis	Color change	(165)
*U. pugilator*	CdCl_2_	10 mg·L^–1^	Reduction of NE-mediated PDH release from SG	Distal pigment migration	(166)
*P. clarkii*	CdCl_2_ HgCl_2_	0.5 µg/g body weight (injection)	Inhibition of 5-HT mediated VSH release	Ovarian growth	(167)
*U. pugilator*	CdCl_2_	1 mg·L^–1^	Increase of VIH secretion from SG	Ovarian growth	(168)
*Chasmagnathus granulata*	CdCl_2_ CuCl_2_	0.5 mg·L^–1^ 0.1 mg·L^–1^	Reduction of VIH secretion from SG	Ovarian growth	(169)
*Palaemon elegans*	CuCl_2_	0.1 + 0.5 mg·L^–1^	5-HT mediated increase of CHH release from SG	Glycaemia	(170, 171)
*Barytelphusa guerini*	DDT	2 mg·L^–1^ (injection)	Increase of CHH release from SG	Glycaemia	(172)
*U. pugilator*	PCB	8 µg·L^–1^ (Aroclor1242)	Reduction of NE-mediated PDH release from SG	Color change	(173)
*Oziotelphusa senex senex*	Fenitrothion	0.1 mg·L^–1^ 0.5, 1, 2 mg·L^–1^	Increase of VIH secretion from SG Increase of CHH release from SG	Ovarian growth Glycaemia	(174) (175)
*U. pugilator*	Naphthalene	10 mg·L^–1^	Inhibition of VSH release	Ovarian growth	(176)
*U. pugilator*	Naphthalene	2.54, 7.83, 9.98 mg·L^–1^	Inhibition of NE-mediated melanin dispersion	Color change	(177, 178)
*U. pugilator*	Fluoxetine Fluvoxamine	20 µg/animal 20 µg/animal	Increased 5-HT mediated red pigment dispersion, reduced red pigment concentration	Color change	(179)
*U. pugilator*	Reserpine Bretylium	20 µg/animal 20 µg/animal	Increased NE-mediated melanin concentration, reduced melanin dispersion	Color change	(180)
*U. pugilator*, *P. clarkii*	Opioids	10^–10^ –10^–8^ mol/animal	Increase of VIH secretion from SG Inhibition of VSH release	Ovarian growth	(181, 182)
*Daphnia magna*	Fluoxetine	40 µg·L^–1^	Increase of offspring production under limiting food conditions *via* 5-HT signaling	Reproductive output	(183)
*Carcinus maenas*	Fluoxetine	0.5 nM (injection)	Stimulation of CHH (and MIH) release from SG	Glycaemia, ecdysteroids	(92)
*C. maenas*	Fluoxetine	0.5–1nM (inject.)	5-HT mediated activation of heart and-scaphognathites	Cardioventilatory activity	(184)
*Erichoeir sinensis*	Carbamazepine	0.01–10 µg·L^–1^	Increase of *chh* and *mih*-expression	Ecdysteroids, moulting	(130)
*Daphnia pulex*	Fluoxetine Citalopram	1 µM 1 µM	Increase of male production under short-day photo-period *via* glutamate/monoamine signaling	Male sex determination	(185)
*Crangon crangon*	Fluoxetine	0.1, 10, 100 ng·L^–1^	Increased 5-HT mediated red pigment dispersion	Color change	(186)

*All concentrations correspond to waterborne exposures unless otherwise stated.

CHH, crustacean hyperglycemic hormone; MIH, moult inhibiting hormone; NE, norepinephrine; PDH, pigment dispersing hormone; SG, sinus gland; VIH, vitellogenesis inhibiting hormone; VSH, vitellogenesis stimulating hormone; 5-HT, 5-hydroxytryptamine, serotonin.

The anti-androgen cyproterone acetate severely reduced growth of *D. magna* without eliciting any discernible effect on moult frequency (145). Effects of several anti-androgens (cyproterone acetate, linuron, vinclozolin, p,p’-DDE) on the reproductive system of copepods revealed varied effects, although consistent among the treatments were degeneration of spermatocytes and deformed spermatophores (163).

#### Evidence Against

##### Lack of a Critical Enzyme for Estrogen Biosynthesis

Aromatase (CYP19) is responsible for the metabolic conversion of androstenedione to estrone and testosterone to estradiol. It is thus critical to the synthesis of estrogens. CYP19 is a product of chordate evolution (187) and has not been detected among the protostome invertebrates. Notably, CYP19 is absent from the *D. pulex* genome (159, 188). While it is possible that estrogens are synthesized in crustaceans *via* an alternative metabolic pathway, we are aware of no support for this premise.

##### Lack of Sex Steroid Receptors

Arguably, the greatest evidence against a role of vertebrate-type sex steroids in crustaceans and other Ecdysozoans is the lack of sex steroid hormone receptors (see Section 2). Immunochemical studies have suggested the presence of estrogen receptor α in *Gammarus fossarum* (27), androgen and estrogen receptors in the mud crab (189), and progesterone and estrogen receptors in crayfish (190). However, immunochemical assays are prone to false positive results due to cross-reactivities or non-specific binding to abundant proteins (191). In the latter studies, putative estrogen receptor co-localized with the other receptor evaluated (progesterone receptor in crayfish and androgen receptor in crab) indicating that antibodies in the same studies may have all been binding to the same abundant protein. Further, results from these studies were inconsistent with estrogen receptor detected in the cytosol from crayfish and membranes of the crab.

We are aware of no reports of the identification of high-affinity sex-steroid binding proteins, indicative of receptors, in crustaceans. Importantly, no sex steroid receptor genes were found in the genome of *D. pulex* (57). Similarly, the sequenced genomes of the ecdyzoans *D. melanogaster* and *Caenorhabditis elegans* revealed no androgen, estrogen, or progesterone receptors [discussed in (27)]. The dominant consensus among researchers is that sex steroid receptors were lost in the lineage leading to the evolution of arthropods (54) and are not present in crustaceans.

##### High Exposure Concentrations of Hormones and EDCs Are Typically Required to Elicit a Response

Steroid, and similar acting, hormones regulate physiological processes such as development, growth, metabolism, and reproduction. Thus, the action of these chemicals, *via* receptor-mediated signal transduction, typically does not result in rapid, overt responses by the organism (*e.g.*, acute responses). Rather, acute responses to these chemicals are largely the consequence of some ancillary response to high exposure concentrations of the chemical (*e.g.*, membrane disruption). In contrast, receptor-mediated responses to the chemical present as long-term consequences, such as alterations in development or reproduction (*e.g.*, chronic responses). These chronic responses are often, though not always, elicited at exposure concentrations significantly below those that elicit acute responses. The magnitude of the difference between concentrations of a chemical that elicit acute *versus* chronic responses is a function, in part, of the binding affinity of the agonist to responsive receptor protein. Hormones bind their receptors with high affinity and, thus, chronic responses to the hormone are typically elicited at concentrations orders-of-magnitude below concentrations that elicit acute toxicity (*e.g.*, high acute/chronic ratio; see **Table 2**, 20E).

**Table 2:** Acute and chronic toxicity values for crustaceans exposed to **(A)** compounds that disrupt ecdysteroid or methyl farnesoate signaling and **(B)** compounds that disrupt estrogen or androgen signaling.

Species	Chemical	EC50 (mg·L^–1^)^1^	MATC (mg·L^–1^)^1^	Acute/chronic ratio	Chronic endpoint
**A. Endocrine active chemicals in crustaceans**					
Water flea (*Daphnia magna*)	Tributyltin	1.67	0.14	11.9	Reproduction
Opossum shrimp (*Americamysis bahia*)	Tributyltin	2.2	0.37	5.9	Growth/reproduction
Water flea (*Daphnia magna*)	Methoprene	340	15.7	21.6	Development
Opossum shrimp (*Neomysis integer*)	Methoprene	320	10	32	Moulting
Water flea (*Daphnia magna*)	Pyriproxyfen	80	0.070	1,143	Reproduction
Opossum shrimp (*Neomysis integer*)	Pyriproxyfen	65	–		
Scud (*Gammarus fossarum*)	Pyriproxyfen	–	1.5	43^2^	Reproduction
Water flea (*Daphnia magna*)	Ponasterone	175^3^	<12.5^3^	>14	Moulting
Water flea (*Daphnia magna)*	20-Hydroxyecdysone	2,457^3^	88^3^	28	Moulting
Water flea (*Daphnia magna*)	Azadirachtin	680	82	8.3	Reproduction
Water flea (*Daphnia magna*)	Tebufenozide	17,370	62	280	Reproduction
Opossum shrimp (*Americamysis bahia*)	Tebufenozide	10,000	138	>72	Growth
**B. Endocrine active chemicals in vertebrates**					
Water flea (*Daphnia magna)*	Bisphenol A	1,336	540	2.5	Reproduction
Water louse (*Asellus aquaticus*)	Bisphenol A	9,500	224	42	Moulting
Water flea (*Daphnia magna)*	Diethylstilbestrol	1,550^4^	350^5^	4.4	Moulting
Water flea (*Daphnia magna)*	4-Nonylphenol	130	35	3.7	Reproduction
Scud (*Hyalella azteca*)	4-Nonylphenol	38	7.0	5.4	Survival
Water flea (*Daphnia magna)*	Atrazine	54,000	6,900	7.8	Reproduction
Copepod (*Amphiascus tenuiremis*)	Atrazine	>1000	86	>11.6	Reproduction
Water flea (*Daphnia magna)*	Cyproterone acetate	–	353^6^	–	Growth/reproduction
Water flea (*Daphnia magna*)	Butyl benzyl phthalate	3,700	444	8.3	Reproduction
Opossum shrimp (*Americamysis bahia*)	Butyl benzyl phthalate	900	113	8.0	Reproduction

^1^Acute (EC50) and chronic toxicity values derived from the EPA EcoTox database (https://cfpub.epa.gov/ecotox/) unless indicated otherwise in comments. Chronic values (CVs) represent the square root of the no observed effect concentration (NOEC) X the lowest observed effect concentration (LOEC)). The NOEC or LOEC was used as a surrogate for the CV where both values were not available. ^2^Value was determined using EC50 with Neomysis and maximum acceptable toxicant concentration (MATC) with Gammarus. ^3^ (192), ^4^ (193), ^5^ (20), ^6^ (145)

Compounds known to be endocrine active in crustaceans typically elicit an acute/chronic ratio of 10–1,000 (**Table 2A**). The chronic responses listed in **Table 2A** are due to disruption of ecdysteroid and MF-signaling. Compounds known to act in vertebrates *via* estrogen and androgen signaling pathways, typically elicit acute/chronic ratios in crustaceans of <10 (**Table 2B**). The latter suggests that chronic responses of crustaceans to these vertebrate EDCs are not elicited through interaction with a hormone receptor.

The strongest evidence for the susceptibility of crustaceans to vertebrate sex steroid agonists are those studies that have shown effects of 17β-estradiol on reproductive system development. Studies cited above reported stimulatory effects of 17β-estradiol on crustacean vitellogenesis (28, 157). However, 17β-estradiol treatments also have been shown to have a negative effect or no effect on crustacean vitellogenesis (194). Exposure of daphnids to the estrogens diethylstilbestrol and bisphenol A had no effect on vitellogenin mRNA levels (195). 17β-Estradiol has been shown to interact with the ecdysteroid receptor at sufficiently high concentrations (196), thus some effects of estrogen injection, the common mode of administration in these studies, may have been the consequence of low affinity interaction of the estrogenic hormone with the ecdysteroid receptor.

## Endocrine Regulation of Color Change

### Neuro-Endocrine Regulation

Active color changes are termed “morphological” in the case of slow color changes established over weeks and months, whereas the rapid type that can take place in minutes to hours is termed “physiological” (197, 198). In arthropods, coloration and reversible color change have evolved together with the development of an exoskeleton (197). Hence, color may be produced by pigments embedded in the pigmented layer of the endocuticle, or by pigment-containing cells in the epidermis (199). Accordingly, morphological color changes are related to moulting, notably, in terms of ontogenetic color changes (200, 201), whereas physiological color changes are produced by chromatophores.

Rapid color changes in most crustaceans rely on the dispersion and aggregation of pigments within stellate cells containing pigment granules. Generally, monochromatic chromatophores, *i.e.*, black-brown melanophores, red erythrophores, yellow xanthophores, and white leucophores, are intimately arranged in clusters called chromatosomes so as to produce a wide variety of colors (197, 198, 202–204). The dispersion of pigment granules, *i.e.*, their migration from the cell center into the ramifications of the chromatophores renders the coloration more intense, whereas the opposite is the case when the pigment granules aggregate within the center of the cell. Dispersion and aggregation of pigment granules can be completed within half an hour in *Crangon* up to 2 h in *Carcinus* or *Macrobrachium* (83, 205–208). Aggregation and dispersion are regulated by an antagonistic system of neuropeptide hormones composed of red pigment concentrating hormone (RPCH, or simply PCH) and pigment dispersing hormone (PDH) with its isoforms α- and β-PDH (198, 203). Notably, RPCH represents a highly conserved neuropeptide, which, in all decapods so far investigated, has an identical sequence (209, 210).

The antagonistic neurosecretory control of rapid color changes belongs to the best-studied hormonal systems in crustaceans [reviewed in (198, 202, 203)]. Indeed, RPCH and PDH have been the first neuropeptides to be characterized in crustaceans (211, 212). It has been established long ago that the eyestalk of decapod crustaceans is the source of hormones regulating color change in crustaceans (202). This was confirmed by Mangerich et al. (213), who localized the main perikarya of RPCH-secreting cells adjacent to or within the XO of *C. maenas* and *Orconectes limosus*, respectively. More recently, Alexander et al. (83) confirmed the presence of about 30 perikarya located in the XO, which project into the SG. A similar situation was shown for PDH, with the majority of PDH-perikarya located between the *medulla interna* and the *medulla lateralis* of the eyestalks of *C. maenas* and *O. limosus* (214), the axon terminals of which may project into the SG. Hence the major neurosecretory structures of RPCH and PDH are located within the eyestalk from where these neurohormones are released into hemolymph that transports them to the respective epithelial target cells. Because pigment dispersion and aggregation in eyestalk ablated animals could be observed, extra-eyestalk sources of RPCH and PDH have been considered. Indeed, RPCH-cells were found in small numbers in the brain, the thoracic ganglia and the circumoesophageal commissure as well as PDH-cells in the thoracic and connective ganglia (83, 213, 215). However RPCH and PDH of some of these cells may rather serve as a neurotransmitter instead of being implied in color change [*e.g.*, (216)].

A model for signal transduction and intracellular signaling cascades upon binding of RPCH to a G-protein coupled receptor has been proposed for the freshwater shrimp *Macrobrachium olfersii* (207, 217). In this model, RPCH activates cyclic guanosine monophosphate (cGMP) and Ca^2+^ second messenger cascades, which in turn stimulate a protein kinase to phosphorylate a myosin II molecular motor. As a result, pigment aggregation is effectuated by the movement of pigment granules along actin filaments in the chromatophore [for details see (198, 218)]. More recently, highly specific RPCH receptors (RPCHR) have been cloned and functionally deorphanized in *D. pulex* and *C. maenas* (83, 210). The RPCHR of *C. maenas* bound RPCH at doses lower than 0.001 nM (EC50 0.02nM). A dose of as low as 0.1 pmol effectively induced pigment aggregation in erythrophores *in vivo* within 5 min and the effect was stronger and longer lasting when the concentrations of RPCH were increased to 1 and 10 pmol, respectively (83). The RPCHR of *D. pulex*, on the other hand, bound *Daphnia*-RPCH at an EC_50_ of 0.065 nM, but binding of crustacean RPCH was at least two orders of magnitude less efficient. Insect adipokinetic hormone did equally activate the *Daphnia*-RPCHR in a dose-dependent and only slightly less efficient manner than *Daphnia*-RPCH (210).

### Potential Sites of Endocrine Disruption

The capacity to change color and to adapt to the surrounding luminance may be impaired by pollutants as different as metals, such cadmium or mercury (165), organic chlorine compounds, like PCBs and naphthalene (173, 177), or drugs that affect the levels of biogenic amines (179, 180, 219) (**Table 1**). In spite of the high concentrations that were employed in these studies, the authors could exclude toxicity and plausibly demonstrate that the respective compounds caused neuroendocrine disruption by affecting the neurotransmitters responsible for the release of, notably, PDH, or the synthesis of the latter. For instance, Aroclor 1242 appeared to reduce norepinephrine (NE) titers in the XO-neuropile (173), thereby reducing the dispersion of black pigment in the chromatophores. Similar observations were made for naphthalene (177). In both studies, the authors took care to verify that neither the chromatophores were affected, which were still able to respond to extracts from the eyestalk containing PDH, nor was the neural tissue damaged. In the case of cadmium, rather the amount of PDH stored in the SG was affected, putatively by inhibiting the synthesis of PDH (165). Fingerman et al. also showed that drugs that deplete monoamine levels, such as reserpine or bretylium, hamper pigment dispersion when fiddler crabs, *Uca pugliator*, were transferred from a white background with concentrated pigments to a black background, whereas fluoxetine enhanced pigment dispersion by increasing 5-HT-levels (179, 180, 219). Therefore, these early studies pointed to the possibility of psychoactive drugs targeting monoamine levels to interfere with the neurohormonal regulation of color change. This was confirmed by recent studies using more environmentally realistic concentrations of waterborne antidepressants (186). Color change in the sand shrimp, *Crangon crangon*, was affected by fluoxetine in the range of 10–1,000 ng·L^−1^ when exposed for 1 day or 1 week (186), suggesting enhanced dark adaptation following fluoxetine exposure (**Table 1**).

## Endocrine Regulation of Sexual Differentiation

Vertebrates typically utilize a variety of genetic sex-determining strategies including sex-determining genes assembled on sex-chromosomes where females are the heterogametic sex, and sex-determining genes assembled on sex-chromosomes where males are the heterogametic sex (220), and autosomal sex-determining genes whose expression are environmentally controlled (221). Common to these sex-determining strategies, sex steroids (androgens, estrogens) are ultimately responsible for sexual differentiation. Indeed, androgens and estrogens from exogenous sources can sometimes circumvent genetic sex-determination (222).

Similarly, crustaceans possess diversity in sex determining mechanisms. Some decapods utilize sex chromosome where female are the heterogametic sex [*Penaeus monodon* (223); *Penaeus japonicus* (224)], while male are the heterogametic sex in others [*Orchestia cavimana* and *Orchestia gammarellus*, (225)]. Among branchiopods, clam shrimp *Eulimnadia texana* consists of monogametic males and heterogametic hermaphrodites (226), and brine shrimp *Artemia franciscana* consist of monogametic males and heterogametic females (227). In contrast, the female and male genomes of *D. pulex* are identical (228). A major distinction between vertebrates and crustaceans is that crustaceans have evolved strategies for sexual differentiation that do not involve steroidal androgens and estrogens.

### Malacostracans

Evidence for an underlying genetic component to sex-determination in malacostracan crustaceans has come from a series of ablation/implantation experiments followed by cross breeding. The chromosomal system for these crustaceans is often referred to as ZW males and WW females. Using isopods (*Armadillidium vulgare*), Suzuki and Yamasaki (229) were able to transform males into functional females through the ablation of the androgenic gland (AG) and females into functional males through the implantation of the AG. The reciprocal crosses of “genetic” males with converted females and “genetic” females with converted females results in single sex broods. Similar experiments have been done with a variety of other crustaceans including prawns, crayfish, and hermit crabs (230–232). Further evidence of a genetic and potential chromosomal basis to sex-determination has come from breeding experiments with intersex crayfish. When intersex crayfish, *Cherax quadricarinatus*, that are functionally males were crossed with females the result was a 1:3 (male:female) sex ratio (232). Subsequent crossbreeding between female (WW) crayfish with normal males resulted in an all-female progeny 0:1 (male:female), leading the authors to conclude that the intersex specimens must have been genetic females (WZ). While the evidence for a genetic component to sex- determination remains strong, sex determination in Crustacea can also show degrees of plasticity. Therefore, it is most likely controlled also by epigenetic factors including environmental variables such as light and temperature (233, 234), parasites (235), and even diet (236).

#### Androgenic Gland Hormone

Male secondary sex characteristics in malacostracan crustaceans are under the control of androgenic gland hormone (AGH), which is produced by the ductless AG (237–239). The AG is usually situated on the paired testes or *vas deferens* in crustaceans. Its important role was discovered through a series of ablation and implantation experiments in the 1950’s (240–243). Removal of AG results in the cessation of spermatogenesis and the demasculinization of male secondary sexual characteristics [(244) and references within]. Complete andrectomy in some species leads to the conversion of testicular to ovarian tissues that have the capacity to accumulate yolk proteins (245). Similarly, implantation of AGs into female crustaceans results in the conversion from ovarian to testicular tissues and the development of male sexual characteristics. Suzuki (230) was also able to demonstrate through a series of these ablation and implantation experiments at different maturation stages within the isopods that AGH was a sex- differentiating, but not a sex-determining factor in these organisms.

First purifications by Hasegawa et al. and Martin et al. have identified the AGH (246–248). The full insulin like peptide structure, consisting of B chain, A chain, and C peptide and the gene sequence of AGH have been characterised in the late 1990s for the isopod *A. vulgare* (249–251). Immunohistochemistry has shown that antibodies raised from AGH-peptides display relatively strong species specificity (252), which is not surprising as sexual characteristics are under strong selection pressures. Unfortunately, this makes developing immuno-histochemistry based bioassays for endocrine disrupter studies more problematic. The cDNA sequence for the insulin-like androgenic gland (IAG) gene has now been reported by several species including crayfish, and several prawn/shrimp and crabs (253). This allows for RNA interference (RNAi) techniques to be used to demasculinize and sex reverse aquaculture species with an AG-specific IAG peptide-encoding transcript (254, 255).

#### Potential Targets of Endocrine Disruption

Currently, it is not known whether environmental pollutants can impact the development of the AG development, or the synthesis of AGH. A number of studies correlated pollutants with increased incidences of intersexuality in crustaceans or male crustaceans displaying certain degrees of feminization or de-masculinization (18). These phenotypic changes in field collected animals mirror the physiological changes caused by feminizing parasite infection, AG ablation or RNAi silencing the AG leading authors to hypothesize whether chemicals can directly or indirectly interfere with the AG or AGH (18, 244). These hypotheses require further testing. In the light of an endocrine axis between the XO/SG, the AG and the male reproductive system, which has been confirmed for decapods (256), a disruption of the neuroendocrine regulation of AGH synthesis and spermatogenesis is conceivable. Indeed, specific CHH-isoforms appear to regulate AGH-expression (257) and its has been demonstrated that metal and organic pollution has the potential to affect CHH-synthesis or -secretion (92, 164, 170–172) (**Table 1**).

### Branchiopods

Sexual differentiation in branchiopods has been extensively studied in *Daphnia*. Daphnids do not possess sex chromosomes (258, 259) and sexual differentiation of offspring is regulated by environmental cues (32). Under environmental conditions that favor rapid population growth, daphnids reproduce parthenogenetically (diploid oocytes) with all offspring being largely female (260). Maternal organisms produce broods, often consisting of dozens of offspring, every few days. These female offspring then mature in a matter of days and begin producing broods of female offspring. As a result, the population expands at an exponential rate. Under conditions that foretell adversity to population sustainability (exhaustion of resources, impending temperature extremes associated with summer or winter), females introduce male offspring to the population. Males mate with females that are producing haploid oocytes (260). The resulting embryo has undergone genetic exchange, which helps to purge deleterious mutations (261). The embryo is in a resting state of diapause to wait out the period of adversity, and is encased in a protective ephippium, which withstands desiccation and freezing. The ephippium is also hydrophobic, which facilitates transport on transient biota (*e.g.*, aquatic birds) or dispersal in air currents. This facilitates dispersal of the organisms to new habitats (262).

#### Environmental Regulation

The role of photoperiod and temperature in male sex differentiation of daphnids has been well characterized. Photoperiod and temperature function in concert to regulate sex ratios in *D. pulex* and *D. magna* populations (263). Under a long-day, summer-like photoperiod, daphnids produced only female offspring, regardless of temperature (range evaluated was 16-22°C). However, under a short-day, autumn-like photoperiod, daphnids became susceptible to temperature-dependent sex-determination. The different species exhibited different temperature optima for male sex-determination, probably relating to the geographic locations at which the populations used in the study were originally derived. Other environmental factors that have been implicated in male sex-determination include food restriction (264) and crowding (265).

#### Neuroendocrine Regulation

Exposure of daphnids to environmental conditions that stimulate the production of male offspring resulted in increased mRNA levels for various components of glutamate signaling based upon gene ontology (GO) terms (266). Further, Camp et al. (185) demonstrated that environmental stimulation of male sex determination resulted in increased mRNA levels of the subunit 2 of the N-methyl-D receptor (NMDAR) while having no effect on the NDMAR-a subunit. This change in the abundance of a single subunit of the receptor would result in alterations in subunit composition of the receptors, which has been shown to be responsible for plasticity in receptor function in vertebrates (267, 268). Conceivable, a reduction in the NMDAR-a/NMDAR-b subunit ratio may prompt glutamate signaling leading to male sex differentiation.

A role for the NMDAR in male sex-differentiation was further indicated by the observation that exposure of maternal daphnids to the NMDAR antagonists MK-801 and desipramine significantly increased the number of male offspring (185). Toyota et al. (266) also observed an effect of MK-801 on male sex differentiation; however, these investigators reported that the NMDAR antagonist suppressed male offspring production (266). Differences in results between these two research teams may reflect differences in experimental design. Where Camp et al. (185) reported the number of male and female offspring produced per female over six broods, Toyota et al. (266) reported the percentage of 30-day-old females that produced males, presumably in a single brood.

MK-801 also inhibits 5-HT, NE, and dopamine reuptake transporters, while desipramine inhibits noradrenergic reuptake transporters (269, 270). Therefore, Camp et al. (185) investigated the potential role of these neurotransmitter-signaling pathways in male sex differentiation. The mRNA levels of the serotonin reuptake transporter *SERT-a* and the α-adrenergic-like octopamine receptor *OctaR-A* were significantly elevated in daphnids reared upon a short-day photoperiod as compared to those reared under a long-day photoperiod. Two selective serotonin reuptake inhibitors fluoxetine hydrochloride and citalopram hydrobrimide increased offspring male sex determination, although the effect of fluoxetine hydrochloride was not statistically significant (p=0.08). These results suggest that in addition to glutamate signaling other neurotransmitters may be operative in male sex differentiation.

#### Methyl Farnesoate

Farnesyl units (C15), derived from acetate serve as building blocks for several important biomolecules, such as cholesterol and steroid hormones. In crustaceans, and other arthropods, farnesyl units also are used for the synthesis of farnesoic acid. Crustaceans utilize farnesoic acid as the substrate for MF. MF is a major sesquiterpenoid hormone in crustaceans, akin to juvenile hormone in insects (271).

Male-sex differentiation depends on MF (272, 273). During late stages of maturation MF programs oocytes to differentiate into males. In the absence of methyl farnesoate, offspring differentiate into females (272). MF is responsible for the induction of the doublesex gene (*dsx1*) during oocyte susceptibility to sex differentiation (274). The doublesex gene product is transcriptionally upregulated in males and is responsible for orchestrating male sex-differentiation (274, 275). Sexually dimorphic expression of the double sex gene also has been shown in other Branchiopod crustaceans including *D. magna*, *Ceriodaphnia dubia*, and *Moina macrocopa* (276).

##### Synthesis and Degradation

In insects, MF is a product of the mevalonate biosynthetic pathway (277), as is likely the case in crustaceans. Two enzymes along this pathway were identified in lobster that were induced commensurate with MF synthesis (278). 3-Hydroxy-3-methyl-glutaryl-coenzyme A reductase (HMGCR) activity increased within 24 h of eye-stalk ablation, which increased MF hemolymph levels, while farnesoic acid O-methyl transferase (FAMeT) activity was increased substantially two weeks after ablation. The reduction of HMGCR is the final step in the biosynthesis of mevalonate; while, methylation of farnesoic acid is the final step in the synthesis of MF. The authors surmised that increased production of mevalonate *via* heightened HMGCR reductase activity was responsible for the immediate increase in MF-production following eyestalk ablation, while prolonged increased synthesis of MF was due to elevation in FAMT activity.

In insects, MF is susceptible to metabolism and inactivation through ester hydrolysis and conjugation to polar molecules (279). These inactivation processes are operative in crustaceans as well (276, 280).

The location of synthesis in branchiopod crustaceans has not been established. In decapod crustaceans, MF is synthesized in the mandibular organ, which is under the negative control of mandibular organ inhibiting hormone (281, 282) (**Figure 1**). MF functions in some aspects of masculinization in decapods, such as the development of the male reproductive morphotype of the spider crab, *Libinia emarginata*. Abraded males (have not moulted in about a year or more) characteristically have high hemolymph MF-levels, large reproductive organs, and aggressive mating behavior (283).

##### Receptor-Mediated Activation

The regulatory activity of MF is mediated primarily through interaction with the bHLH-PAS protein methoprene-tolerant (MET) (284, 285). MET derives its name from the discovery that resistance of *Drosophila* to the insecticidal MF-analog, methoprene, was associated with a functional mutation in this gene (286). MF-activated MET recruits the bHLH-PAS protein, steroid receptor coactivator (SRC) (37). Together, this MET-SRC complex comprises the activated MF- receptor (MfR) in crustaceans.

Other suggested receptors for MF in crustaceans include the RXR and hormone receptor 97g (HR97g). RXR is a member of the nuclear receptor superfamily that has been identified in several crustacean species including the American lobster *Homarus americanus* (287), the fiddler crab *Uca pugilator* (288), the tropical land crab *Gecarinus lateralis* (289), the crayfish *Procambarus clarkii* (290), the amphipod *G. fossarum* (291) and the water fleas (*D. magna, D. pulex*) (58, 292). While MF was unable to activate daphnid RXR in a luciferase reporter assay, its co-administration with 20E to a reporter system consisting of RXR and EcR, resulted in activation greater than that observed with 20E alone (293). This apparent synergistic interaction between activated EcR and activated RXR was also observed *in vivo* using tributyltin as the RXR agonist (294). These results suggest that MF, through interaction with RXR, may function in concert with 20E to regulate crustacean moulting.

Nuclear receptor HR97g, isolated from *D. pulex*, was mildly activated by MF and the MF-analog pyriproxyfen in a luciferase reporter assay (295). This crustacean receptor was first identified in *D. pulex* (57), and has since been identified in the spiny lobster *Panulirus ornatus* (296). The physiological significance of the receptor as a ligand-activated regulator of crustacean physiology remains unknown.

#### Known Targets of Disruption

The MfR is the best-demonstrated target of disruption of this regulatory pathway by environmental chemicals. Compounds that elicit insecticidal activity as juvenile hormone analogs also typically bind and activate the MfR (37, 285). This activity is responsible for the high sensitivity of crustaceans to this class of insecticides (273, 297, 298). However, the MfR appears to have high ligand recognition specificity. We are aware of no demonstrations that compounds, other than juvenile hormone analogs, are capable of activating the MfR at environmentally relevant exposure levels (37).

#### Potential Targets of Disruption

Alterations in MF levels due to toxicant-mediated effects on biosynthetic or inactivating hormones is a plausible mechanism of endocrine disruption. The herbicide atrazine reportedly increased male offspring production in *Daphnia pulicaria* (299). However, atrazine did not interact with the MfR (37), suggesting that if atrazine did indeed activate this pathway, it may have been due atrazine increasing endogenous MF. Such an effect may have been the consequence of competition between atrazine and MF at a site of inactivation or elimination. Alternatively, atrazine disrupts endocrine function in mammals by increasing dopamine and reducing NE levels in the hypothalamus (300). These neuromediators are operative in the regulation of MF and sex determination in daphnids (185). Thus, disruption of these upstream signaling components may be responsible for the effects of atrazine on the MF-signaling pathway.

## Future Directions of Research on Endocrine Disruption in Crustaceans

In 1998, a workshop was held in the Netherlands to address the issue of endocrine disruption in invertebrates sponsored by The Society of Environmental Toxicology and Chemistry (SETAC). The proceedings of this workshop were subsequently published (301) and made a number of recommendations. Recently, Ford and LeBlanc conducted a survey of experts to reflect on the progress made in endocrine disruptor studies with invertebrates over the past few decades (302). The majority of participants in that survey believed endocrine disruption was an issue for invertebrates that needed to be addressed, but were mixed over the relative progress that had been made. Strikingly, many of the recommendations provided in this recent survey mirrored those made back in 1998 indicating that the field had not significantly progressed.

A major impediment to advancing research on endocrine disruption in crustaceans has been attempts to detect endocrine effects using chemicals known to be endocrine disruptors in vertebrate species along with endpoints known to be indicative of endocrine disruption in vertebrates. This conclusion has recently been acknowledged by several researchers (303, 304). For example, many studies focus upon the effects of estrogens on crustaceans (18) and use biomarkers of feminization, such as vitellogenin induction, in crustaceans (305). Elevated vitellogenin levels following chemical exposure or in field-collected samples has often been interpreted as an estrogenic effect (306). However, unlike vertebrates, vitellogenin is not regulated by estrogens in crustaceans (195). Despite existing knowledge on the endocrine regulation of vitellogenesis in crustaceans, the precise molecular mechanisms by which vitellogenesis and ovarian maturation are controlled require further elucidation (307). Given the lack of clear understanding of seasonal or developmental fluctuations in normal vitellogenin levels and overall susceptibility of vitellogenin production to non-endocrine stressors, associations between altered vitellogenin levels and endocrine disruption in crustaceans are tenuous at best.

One area where progress has been made is the onset of more affordable “omic” technologies allowing for the high-throughput sequencing of genomes, transcriptomes, peptidomes, and metabolomes (19, 139, 304). These omics-techniques offer a rich opportunity for discovering conserved molecular and biochemical pathways, which can applied to the development of adverse outcome pathways (308). These in turn provide opportunities for further advancing our mechanistic understanding of crustacean endocrinology from which to develop appropriate biomarkers of disruption. Zou (139) in a recent review identified over 30 compounds that have either inhibited or stimulated moulting in crustaceans. However, in the absence of mechanistic linkages between exposure and effect, endocrine disruption cannot be invoked as responsible for these effects on moulting. The identification of appropriate biomarkers will facilitate establishing whether an effect on moulting, or some other endocrine-regulated process, is due to specific disruption of the endocrine system or due to some non-specific toxicity.

We hope to have advocated strongly that a “crab is not a fish” and therefore toxicity evaluations using crustaceans require appropriate endpoints to determine whether current and newly licensed chemical compounds might target endocrine processes in this ecologically important group. While significant progress has been made in our understanding of crustacean endocrinology, application of this knowledge to the study of endocrine disruption in crustaceans is lagging. Where, in the past, we were limited in our ability to develop the tools to confirm whether a substance was an endocrine disruptor, these limitations have been largely overcome with affordable omics-technologies. We now have the ability to develop high throughput screenings using key crustacean-relevant endocrine targets. Given the number of crustacean species incorporated into national toxicity programs, such tools are sorely needed.

## Author Contributions

TK, GL, and AF have developed the concept of this review and have contributed to the writing. All authors contributed to the article and approved the submitted version.

## Funding

This work was financially supported by the European Union through the Interreg France (Channel) England program. TK and AF worked on this article in the framework of the Project “Reduction of Pollution by endocrine disrupting compounds at source: innovative products for the commercial lab market” (RedPol; Interreg 5 a, # 185).

## Conflict of Interest

The authors declare that the research was conducted in the absence of any commercial or financial relationships that could be construed as a potential conflict of interest.

## Abbreviations

AG, androgenic gland; AGH, androgenic gland hormone; CCAP, crustacean cardioactive peptide; CHH, crustacean hyperglycemic hormone; Cyp19, aromatase; EcR, ecdysone receptor; EDC, endocrine disrupting chemical; EH, eclosion hormone; ETH, ecdysis triggering hormone; FAMeT, farnesoic-O-methyl transferase; HMGCR, 3-hydroxy-3methyl-glutaryl coenzyme A reductase; IAG, insulin-like androgenic gland factor; MET, methoprene tolerant; MF, methyl farnesoate; MfR, methyl farnesoate receptor; MIH, moult inhibiting hormone; NE, norepinephrine; NMDAR, N-methyl-D-aspartate receptor; PCBs, polychlorinated biphenyls; PDH, pigment dispersing hormone; PoA, ponasterone; RPCH, red pigment concentrating hormone; RPCHR, red pigment concentrating hormone receptor; RNAi, RNA interference; RXR, retinoid-X-receptor; SRC, steroid receptor coactivator; US EPA, United States Environmental Protection Agency; XO/SG, X-organ/sinus gland; 5-HT, 5-hydroxytryptamine, serotonin; 20E, 20-hydroxyecdysone.
